# Products purchased from family farming for school meals in the cities of Rio Grande do Sul

**DOI:** 10.1590/S1518-8787.2017051006648

**Published:** 2017-02-08

**Authors:** Daniele Ferigollo, Vanessa Ramos Kirsten, Dienifer Heckler, Oscar Agustín Torres Figueredo, Julian Perez-Cassarino, Rozane Márcia Triches

**Affiliations:** I Curso de Nutrição. Universidade Federal de Santa Maria. Palmeira das Missões, RS, Brasil; II Departamento de Alimentos e Nutrição. Universidade Federal de Santa Maria. Palmeira das Missões, RS, Brasil; III Curso de Engenharia Florestal. Universidade Federal de Santa Maria. Frederico Westphalen, RS, Brasil; IV Departamento de Engenharia Florestal. Universidade Federal de Santa Maria. Frederico Westphalen, RS, Brasil; V Programa de Pós-Graduação em Agroecologia e Desenvolvimento Rural Sustentável. Universidade Federal da Fronteira Sul. Laranjeiras do Sul, PR, Brasil

**Keywords:** School Feeding, Food Supply, Urban Agriculture, Food and Nutrition Security

## Abstract

**OBJECTIVE:**

This study aims to verify the adequacy profile of the cities of the State of Rio Grande do Sul, Brazil, in relation to the purchase of products of family farming by the *Programa Nacional de Alimentação Escolar* (PNAE - National Program of School Meals).

**METHODS:**

This is a quantitative descriptive study, with secondary data analysis (public calls-to-bid). The sample consisted of approximately 10% (n = 52) of the cities in the State, establishing a representation by mesoregion and size of the population. We have assessed the percentage of food purchased from family farming, as well as the type of product, requirements of frequency, delivery points, and presence of prices in 114 notices of public calls-to-bid, in 2013.

**RESULTS:**

Of the cities analyzed, 71.2% (n = 37) reached 30% of food purchased from family farming. Most public calls-to-bid demanded both products of plant (90.4%; n = 103) and animal origin (79.8%; n = 91). Regarding the degree of processing, fresh products appeared in 92.1% (n = 105) of the public calls-to-bid. In relation to the delivery of products, centralized (49.1%; n = 56) and weekly deliveries (47.4%; n = 54) were the most described. Only 60% (n = 68) of the public calls-to-bid contained the price of products.

**CONCLUSIONS:**

Most of the cities analyzed have fulfilled what is determined by the legislation of the PNAE. We have found in the public calls-to-bid a wide variety of food, both of plant and animal origin, and most of it is fresh. In relation to the delivery of the products, the centralized and weekly options prevailed.

## RESUMO

**OBJETIVO:**

Verificar o perfil de adequação dos municípios do Rio Grande do Sul no que tange à aquisição de produtos da agricultura familiar pelo Programa Nacional de Alimentação Escolar.

**MÉTODOS:**

Trata-se de estudo quantitativo descritivo, com análise de dados secundários (chamadas públicas). A amostra foi composta por aproximadamente 10% (n = 52) dos municípios do estado, tomando-se o cuidado de estabelecer uma representatividade por mesorregião e tamanho da população. Foi avaliado o percentual destinado às compras de gêneros alimentícios da agricultura familiar, bem como o tipo de produto, exigências de periodicidade, pontos de entrega e presença de preços em 114 editais de chamadas públicas, no ano de 2013.

**RESULTADOS:**

Dos municípios analisados, 71,2% (n = 37) atingiram 30% de gêneros alimentícios oriundos de agricultura familiar. A maioria das chamadas públicas demandou tanto produtos de origem vegetal (90,4%; n = 103) como de origem animal (79,8%; n = 91). Quanto ao grau de processamento dos alimentos, os produtos *in natura* apareceram em 92,1% (n = 105) das chamadas públicas. Em relação à entrega dos produtos, a centralizada (49,1%; n = 56) e as entregas semanais (47,4%; n = 54) foram as mais descritas. Apenas 60% (n = 68) das chamadas públicas continham preço dos produtos.

**CONCLUSÕES:**

A maioria dos municípios analisados cumpriu o determinado pela legislação do Programa Nacional de Alimentação Escolar. Encontrou-se nas chamadas públicas uma grande diversidade de alimentos, tanto de origem vegetal quanto de origem animal, sendo a maior parte deles *in natura*. Em relação à entrega dos produtos, prevaleceu a centralizada e a semanal.

## INTRODUCTION

The *Programa Nacional de Alimentação Escolar* (PNAE - National Program of School Meals) is one of the oldest permanent federal government interventions on food supplementation in the context of social and welfare policies in Brazil^12^. Moreover, it is one of the largest programs of school meals in the world, being free and covering the entire country^a^, considered today as an important strategy of *Segurança Alimentar e Nutricional* (SAN - Food and Nutritional Security)^10,12,13,24,b^.

Until 1994, the design, management, and acquisition of food by the PNAE were centralized, by public bidding, and distributed throughout the country^14^. From that same year, several advances happened, such as the decentralization of resources and greater participation of civil society in the management of the program^12^. In 2003, with the *Programa Fome Zero* (Zero Hunger Program) and discussions about the policies of SAN, the PNAE was revised, increasing the federal resources allocated and the public served^14^. Additionally, Resolution 32 of August 10, 2006 enacted, among its guidelines, the support for sustainable development, the promotion of healthy and adequate diet, and the education on food and nutrition^c^.

Law No. 11,947 was approved on June 16, 2009, which provides for the school food service, among its objectives. This contributed to the formation of healthy eating habits at school by enforcing the use of, at least, 30% of the total financial resources transferred by the *Fundo Nacional de Desenvolvimento em Educação* (FNDE - National Education Development Fund) to States and cities for the purchase of food from family farming^d^.

From this law, intersectoriality is strengthened^13^, communities are developed in an economic and sustainable way^3,14,15,17^, and social inequality^15^, poverty and field-city migration are reduced^9,10^ from the redistribution of income to family farmers, thus contributing to the promotion of the SAN and food sovereignty of Brazil^9^. Furthermore, a study in the South region of Brazil has shown that this law also changed the acceptance of healthier and suitable food by the students^14^.

Another innovation of this law relates to the law on public procurement, as the supplier characterized as “family farmer” does not need to go through a bidding process^14^. Currently, the law provides for the use of public calls-to-bid for the purchase of food from family farmers for school meals^e^.

Previous experiments carried out in Brazilian cities indicate that, although in the last decade SAN policies have supported the construction of short supply chains, this law is difficult to be implemented. The highlights are: difficulties of farmers to organize and plan, logistics problems and costs, lack of financial and managerial training of the players involved or understanding of the possibilities engendered by the public policy, lack of documents and interest from the farmers, their distrust in relation to public administration, absence of family farming in the region, infeasibility of regular and constant supply, and lack of collaboration between managers and farmers^1,8,10,11^.

However, to date, few studies have investigated situational diagnoses regarding the process of implementation of local purchases by the PNAE, as the law is relatively recent and the process of implementation is still in the beginning in several cities of the country^17^.

In this sense, it is important the development of more research studies to assess this implementation, considering the need to strengthen and encourage family farming and encourage the use of food that meet local habits and increase consumption of fresh food by students, as defined in the guidelines of the PNAE^d^.

Given these considerations, the objective of this study was to verify the adequacy profile of the cities of Rio Grande do Sul regarding the purchase of family farming products by the PNAE, as well as analyze the public calls-to-bid regarding the characterization of these products, the frequency of receipt, and the delivery points by the farmers.

## METHODS

This is a quantitative descriptive study, with secondary data analysis (public calls-to-bid) of local governments of the cities of Rio Grande do Sul (RS), Brazil. The research was carried out from July to December, 2014.

The study had as reference the methodology proposed by Baccarin et al.^1,2^, described in detail below.

For data analysis, approximately 10.0% of the 497 cities of RS were selected by lottery, establishing a representation by mesoregion and population size. The population size of the cities was classified as: very small (less than 20,000 inhabitants), small (from 20,000 to 100,000 inhabitants), average (from 100,000 to 500,000 inhabitants) and large (over 500,000 inhabitants). The mesoregions were: Western Center, Eastern Center, Metropolitan of Porto Alegre, Northeast, Northwest, Southeast, and Southwest (Figure).

**Figure:**
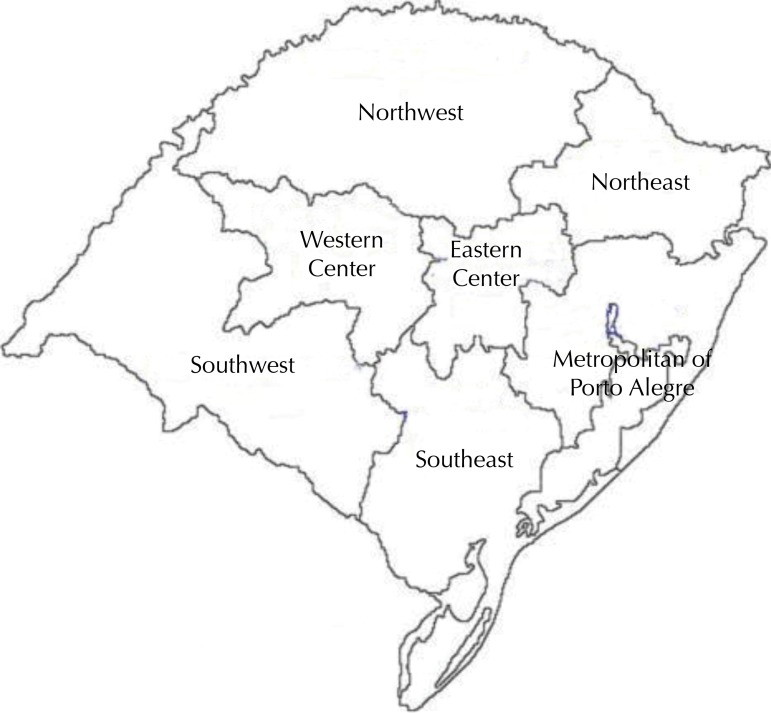
Map of the location of the mesoregions of Rio Grande do Sul (RS), Brazil.

After selecting the cities, we analyzed the official documents of 2013, such as public calls-to-bid in the websites of the local governments and municipal public transparency portals. However, when the information was not available in the websites, we called the local governments.

Of the selected documents, we expected to obtain the following data:

- number of local governments that meet what is determined by Article 14 of Law No. 11,947/2009.
- percentage for the purchase of products of family farming. We highlight that we have collected this information by calling qualified professionals of local governments, such as, for example, the nutritionists responsible for the sector.
- type of product purchased, specifying if it is of plant or animal origin and its degree of processing.
- requirements regarding frequency and delivery points.
- frequency of the product in public calls-to-bid.

For the level of service provided for in Law No. 11,947/2009, the local governments were classified according to the following levels: no service, partial service (spending less than 30% with products of family farming), legal service (spending between 30% and 40% with products of family farming), and differentiated service (spending above 40% with products of family farming).

Regarding the type of product purchased, in addition to seeing if it was of animal or vegetable origin, we considered the following levels of processing:

- Fresh products, which do not undergo any degree of processing;
- Medium degree of processing for food that can be processed and cleaned by the farmer, considering the legal specifications in force;
- High degree of processing for products that necessarily require industrial processing beyond the family farming or its organizations^7^.

The delivery and frequency of products in the public calls-to-bid were classified as: two to five times a week, weekly, three to five times a month, once or twice a month, some times a year, and no information available. In relation to delivery points, they were classified as: very decentralized, with more than fifty receiving units; decentralized, between eleven and fifty receiving units; little decentralized, between two and ten receiving units; and centralized, with a single receiving unit.

In most public calls-to-bid, a factor does not exclude the presence of another. For example, the same notice may request fresh products and also products with a high degree of processing. It may also require different deadlines in the delivery schedule: weekly, twice a week, or even every other week. Therefore, the total values found usually exceed the number of notices analyzed.

## RESULTS

The research was carried out with fifty-two cities of the State of Rio Grande do Sul, Brazil. The number of notices of public calls-to-bid assessed ranged from one to ten by city, amounting to 114 notices.

The region with the highest percentage of cities contemplated was the Northwest, amounting to 23.1% (n = 12) of the cities. The metropolitan region of Porto Alegre amounted to 17.3% (n = 9). The percentages of the other regions were: Southeast – 15.4% (n = 8), Western Center – 13.5% (n = 7), Southwest – 11.5% (n = 6), Eastern Center – 9.6% (n = 5), and Northeast – 9.6% (n = 5). Half of the cities assessed (50%; n = 26) were very small, 36.5% (n = 19) were small, 11.5% (n = 6) were average, and only one (2%) was large.

Among the 52 cities selected for the research, most (71.2%; n = 37) reached 30% or more of the total financial resources transferred by the FNDE for the purchase of food directly from family farming, thus meeting what is provided for in Article 14 of Law No. 11,947/2009.

By stratifying these data, we can observe that 42% (n = 22) of the cities analyzed had differentiated service level and 31% (n = 16) had legal service level. In contrast, 4% (n = 2) of the cities did not buy food from family farmers for school meals and 23% (n = 12) bought, but did not reach the minimum of 30% required by law.

According to Table 1, most notices of public calls-to-bid demanded both plant and animal products, being most of them fresh. Regarding the logistical aspects present in the notices, the request for delivery in a single receiving unit (for example, in the sector of school meals) was prevalent. In relation to the frequency of deliveries, the most frequent were weekly and once or twice a month. However, 21.9% of the public calls-to-bid did not define the frequency. Only 60% of the public calls-to-bid contained the price of products.

**Table 1:** Profile of notices of public calls-to-bid for family farmers for school meals of cities of the State of Rio Grande do Sul, Brazil, 2013. (N = 52)

Variable	n	%
Origin of food		
Animal	91	79.8
Plant	103	90.4
Degree of food processing		
Fresh	105	92.1
Average	103	90.4
High	82	71.9
Frequency of delivery		
2 to 5 times/week	8	7.0
Weekly	54	47.4
3 to 5 times/month	2	1.8
once or twice/month	43	37.7
Sometimes/year	6	5.3
No information available	25	21.9
Receiving Units (RU)		
Centralized (1 RU)	56	49.1
Little decentralized (between 2 and 10 RU)	20	17.5
Decentralized (11 and 50 RH)	28	24.6
Very decentralized (more than 50 UR)	2	1.8
Contains the prices of products		
Yes	68	60.0
No	46	40.0

Total	114	100

Source: Notices of public calls-to-bid of the local governments of the cities selected (2013).

Table 2 lists the food asked from the family farmers. In the group of fruits, orange had the highest percentage, followed by banana, bergamot orange, apple and strawberry. Juices and fruit pulp appeared in 14% and 7.9% of the calls-to-bid, respectively. Among the vegetables analyzed, the most present were: cabbage, lettuce, carrots and beets (64.9%; n = 74). Regarding the legumes, beans were in 44.7% of the calls-to-bid.

**Table 2:** Food purchased from family farms by the cities of the State of Rio Grande do Sul, Brazil, for school meals, 2013.

Variable	n	%
Fruits		
Orange	54	47.4
Banana	28	24.6
Bergamot orange	28	24.6
Apple	27	23.7
Strawberry	24	21.1
Vegetables		
Cabbage	83	72.8
Lettuce	80	70.2
Carrot	75	65.8
Beet	74	64.9
Chives and parsley	61	53.5
Collard greens	59	51.8
Tomato	54	47.4
Legumes		
Beans	51	44.7
Green beans	18	15.8
Lentils	3	2,6
Peas	2	1.8
Cereals, roots and tubers		
Rice	45	39.5
Cookies	30	26,3
Sweet potato	60	52.6
Potato	46	40.4
Cassava	53	46.5
Sugar and preserves		
Dulce de leche	33	28.9
Honey	20	17.5
Molasses	15	13.2
Brown sugar	12	10.5
Jam	10	8.8
Milk and dairy products		
Milk beverage	38	33.3
UHT milk	31	27.2
Powdered milk	27	23.7
Cheese	18	15.8
Meat and eggs		
Chicken (drumstick and thigh)	40	35.1
Chicken egg	27	23.7
Pork	19	16.7
Beef	18	15.8
Chicken breast	18	15.8

Source: Notices of public calls-to-bid of the local governments of the cities selected (2013).

The most frequent cereals, tubers and roots were: rice, sweet potato, potato and cassava. As for sugars and sweets, dulce de leche and honey were the most frequent. Amongst milk and dairy products, milk beverage appeared in 33.3% of the calls-to-bid, followed by whole UHT milk. Regarding meats and eggs, chicken meat - drumstick and tight - appeared the most (35.1%), and chicken egg was present in 23.7% of the calls-to-bid.

## DISCUSSION

Considering that the implementation of Law No. 11,947/2009 is relatively recent, this study aimed to characterize the selected cities of the State of Rio Grande do Sul regarding the purchase of food from family farmers for school meals in 2013.

In Rio Grande do Sul, this type of research is innovative in the analysis of the origin and the degree of processing of products demanded by the notices of public calls-to-bid, as well as the requirements related to frequency, delivery points, and product prices. Moreover, we have identified the percentage of products purchased from family farming by the cities. Baccarin et al.^1,2^ have similarly analyzed the adequacy of public calls-to-bid and product availability in the State of São Paulo, Brazil.

This study has found a high percentage (above 70%) of cities in Rio Grande do Sul meeting what is provided for in Article 14 of Law No. 11,947/2013. Other studies have also proved successful situations regarding the purchase from family farmers for school meals in cities in the State of Rio Grande do Sul, such as Dois Irmãos, which used approximately 60% of the total federal resources for the acquisition of products of family farming in 2009^14^, and São Lourenço do Sul, which used 51.5% in 2010^13^.

When we compare the data from this study and the study of Saraiva et al.^10^, who have assessed the first year of compulsory purchase of family farming in several Brazilian cities, approximately half of the cities assessed (47.4%) had already purchased food from family farming for the PNAE and the percentage of this purchase was, on average, 22.7%. The data from this study corroborate with those of Saraiva et al.^10^, in which the South region of Brazil presented the highest percentage of food purchased from family farming (71.3%). In 2012, the proportion of these purchases in Brazil increased to 67%, and it reached 87% in the South region, in view of the high level of physical and social capital, including the high levels of cooperative affiliation^f^.

According to Saraiva et al.^10^, the South stands out in the production of family farming and in the internal supply of food, in addition to being a significant contributor to the Gross Domestic Product^g^, which may justify the data on the purchase of family farming for the school meals in this region^10^.

Given the requirement of the law for the purchase of basic, diversified, and highly nutritional products, it is essential the survey of the products purchased from family farmers by the notices of public calls-to-bid for school meals^d^. In this research, we can say that there was a demand for diversified food and products of high nutritional value by the cities of the State of Rio Grande do Sul, given the wide variety of food products found and the high percentage of fresh and minimally processed products, such as fruits and vegetables.

It is worth noting that many items that were once produced only for the consumption of farmers now guarantee the market and income of rural families who supply the PNAE, concurrently improving the food and nutrition quality of schools. The budget of the program was R$3.5 billion in 2014, which benefited forty-three million students of basic education. With Law No. 11,947/2009, 30% of this amount (i.e., R$1.05 billion) should be invested in the direct purchase of products of family farming^a^.

By analyzing the profile of the origin of the food, we could see a high demand for both plant and animal products. On the other hand, Baccarin et al.^2^, in the State of São Paulo, verified a high percentage (93.7%) of plant products and less than half (41.6%) of animal products. The data of this research suggest that farmers are adapting to the health law and the issues of prior industrial processing of animal products, usually carried out in large units. It is important to point out that these issues were difficulties faced by family farmers^1,14^.

In this study, fresh products were predominant in the public calls-to-bid. A similar result has been found by Baccarin et al.^2^, who show that fresh products were present in 81.2% of the public calls-to-bid. Such a result is positive because it encourages a healthy and proper diet, which comprises the use of varied and safe food, as well as the preparation of school meals using basic food, in conformity with the nutritional references and the local eating habits, also being guided by sustainability and agricultural diversification in the region^13^.

Regarding the delivery of the products, the public calls-to-bid of almost half of the sample chose a single unit, i.e. it was centralized, which is similar to that found by Baccarin et al.^2^. This facilitates the work of farmers, as they manage to fulfill their responsibilities, better plan the logistical and transport issues, and meet the food distribution of the schools in the city and region.

Most of the deliveries in the public calls-to-bid were weekly (47.4%). The every other week or monthly delivery also had its relevance (37.7%), a result that differs from that found by Baccarin et al.^2^, in which 66.7% of the calls-to-bid demanded weekly deliveries and 33.3%, every other week or monthly. We can say that the more detailed the delivery schedule, the better the planning of farmers regarding their obligations and reduced costs of transportation^2^.

Regarding prices, we have found that not all public calls-to-bid contained this information. This datum is extremely important, since the purchases of these products, using public calls-to-bid, should have a price list. The presence of this information ensures that prices are not higher than those sold as wholesale or retail, not burdening the public purchases. In addition, it ensures better remuneration of family farmers than what they obtain in conventional sales channels^d^.

It is important to note that products purchased from family farming for school meals provide a greater supply of fresh or minimally processed food, being of known origin and therefore more reliable. They, thereby, meet the Brazilian food guide^5,6^ and assist in the formation of more appropriate eating habits, considering the agricultural options and food culture of the region and contributing to the growth, development and improvement of the academic achievement of students.

Thus, the stimulus and support to family farming have been shown to be relevant for the formulation and implementation of municipal efforts of SAN and for local development aimed at promoting the human right to a proper diet^4,16^. That is why, food production, especially from family farming, has been strengthened with initiatives for collaboration by public policies, such as the PNAE^10^.

These policies, therefore, become relevant for the issue of food and nutritional security, impacting on health and environment. The production modes have been improving, such as the encouraging of the production and supply of organic products and the expansion of institutional markets.

It is important to point out that this study presents possible limitations, since not all products asked in public calls-to-bid may have been actually purchased because of seasonality, losses because of droughts, rains, and intense cold, as well as because some farmers stopped delivering.

We ratify that menus elaborated for school meals must be flexible and the decision-making process must be based on the dialog between the need of a product and the delivery availability. We suggest the creation of a network of contact and dialog between the producer (who supplies the food) and the manager (who elaborates the menu) to establish a relationship that is committed, not only to the automatic gesture of receiving and delivering, but to the solid construction of these institutional markets.

Moreover, as limitations of this study, we include the difficulties of obtaining official documents on the websites of local governments – most were obtained by direct contact (via phone) – as well as the lack of access to the accounts of the cities during the period of research.

Furthermore, in relation to the number of cities researched, although the sample had regional representation, approximately only 10% of the cities of Rio Grande do Sul were present, and, in this way, caution must be taken when extrapolating the data for the entire State.

Therefore, we suggest the expansion of the study to more cities of Rio Grande do Sul and other Brazilian States, in view of the importance of food from family farming for school meals, to assess the implementation and effectiveness of the PNAE. The knowledge of the data about the purchase of products of family farming for school meals in other States could contribute both for the local economic development and for the provision of meals to students that meet the principles of a healthy and proper diet.

In conclusion, the results of this study showed that most of the selected cities of the State of RS reached 30% or more of total financial resources transferred by the federal Government for the purchase of food of family agriculture, pursuant to legislation of the PNAE. Moreover, we have found in the public calls-to-bid a wide variety of food, both of plant and animal origin, and most of it was fresh. In relation to the delivery of the products, the centralized and weekly options prevailed.

Although the data presented are still initial, we can see that the PNAE is able to imbue new uses on the territory, pointing socio-cultural, economic, and environmental reflections on the quality of life of family farmers and the students benefited from the program.

## Footnotes

a Ministério da Educação (BR), Fundo Nacional de Desenvolvimento da Educação. Alimentação escolar: Programa Nacional de Alimentação Escolar PNAE. Brasília (DF); 2009 [cited 2015 Mar 4]. Available from: http://www.fnde.gov.br/programas/alimentacao-escolar

c Ministério da Educação (BR), Fundo Nacional de Desenvolvimento da Educação, Conselho Deliberativo. Resolução/ CD/FNDE/ Nº 32, de 10 de agosto de 2006. Estabelece as normas para a execução do Programa Nacional de Alimentação Escolar - PNAE. Brasília (DF); 2006 [cited 2015 Mar 4]. Available from: ftp://ftp.fnde.gov.br/web/resolucoes_2006/res032_10082006.pdf

d Ministério da Educação (BR), Fundo Nacional de Desenvolvimento da Educação, Conselho Deliberativo. Lei Nº 11.947, de 16 de junho de 2009. Dispõe sobre o atendimento da alimentação escolar e do Programa Dinheiro Direto na Escola aos alunos da educação básica. *Diario Oficial Uniao.* 20 set 2009; Seção 1: 2.

e Ministério da Educação (BR), Fundo Nacional de Desenvolvimento da Educação, Conselho Deliberativo. Resolução/CD/FNDE nº. 38, de 16 de julho de 2009. Dispõe sobre o atendimento da alimentação escolar aos alunos da educação básica no Programa Nacional de Alimentação Escolar- PNAE. Brasília (DF); 2009 [cited 2015 Mar 4]. Available from: https://www.fnde.gov.br/fndelegis/action/UrlPublicasAction.php?acao=abrirAtoPublico&sgl_tipo=RES&num_ato=00000038&seq_ato=000&vlr_ano=2009&sgl_orgao=CD/FNDE/MEC

f Soares FV, Nehring, Schwengber RB, Rodrigues CG, Lambais G, Balaban DS, et al. Structured demand and smallholder farmers in Brazil: the case of PAA and PNAE. Brasília (DF): IPC/WFP; 2013 [cited 2015 Apr 24]. Available from: http://www.ipc-undp.org/pub/IPCTechnicalPaper7.pdf

g Guilhoto JJM, Ichihara SM, Silveira FG, Diniz BPC, Azzoni CR, Moreira GRC. A Importância da agricultura familiar no Brasil e em seus estados. São Paulo: Faculdade de Economia e Administração da USP, Fundação Instituto de Pesquisas Econômicas (FIPE); 2007 [cited 2015 Apr 24]. Available from: htpp://www.usp.br/feaecon/media/livros/file_459.pdf
